# Identification of microRNAs involved in acute rejection and spontaneous tolerance in murine hepatic allografts

**DOI:** 10.1038/srep06649

**Published:** 2014-10-17

**Authors:** Miwa Morita, Jiajie Chen, Masayuki Fujino, Yusuke Kitazawa, Atsushi Sugioka, Liang Zhong, Xiao-Kang Li

**Affiliations:** 1 Division of Transplantation Immunology, National Research Institute for Child Health and Development, Tokyo; 2 Department of Surgery, Fujita Health University School of Medicine, Aichi, Japan; 3 Department of Gastroenterology, Huashan Hospital, Fudan University, Shanghai, China; 4 AIDS Research Center, National Institute of Infectious Diseases, Tokyo, Japan

## Abstract

Graft acceptance without the need for immunosuppressive drugs is the ultimate goal of transplantation therapy. In murine liver transplantation, allografts are accepted across major histocompatibility antigen complex barriers without the use of immunosuppressive drugs and constitute a suitable model for research on immunological rejection and tolerance. MicroRNA (miRNA) has been known to be involved in the immunological responses. In order to identify mRNAs in spontaneous liver allograft tolerance, miRNA expression in hepatic allografts was examined using this transplantation model. According to the graft pathological score and function, miR-146a, 15b, 223, 23a, 27a, 34a and 451 were upregulated compared with the expression observed in the syngeneic grafts. In contrast, miR-101a, 101b and 148a were downregulated. Our results demonstrated the alteration of miRNAs in the allografts and may indicate the role of miRNAs in the induction of tolerance after transplantation. Furthermore, our data suggest that monitoring the graft expression of novel miRNAs may allow clinicians to differentiate between rejection and tolerance. A better understanding of the tolerance inducing mechanism observed in murine hepatic allografts may provide a therapeutic strategy for attenuating allograft rejection.

Organ transplantation is a well-known treatment for end-stage organ failure. The development of advanced surgical techniques and immunosuppressive drugs has led to improvements in short-term outcomes after transplantation. However, recipients must receive life-long immunosuppressive drugs, the side effects of which can cause carcinogenesis and infectious disease. In human liver transplantation, it has been reported that some recipients have been weaned off immunosuppressive drugs^1^,^2^, and some experimental transplantation models have shown that liver allografts can be accepted without the need for immunosuppression^3^,^4^,^5^,^6^,^7^. However, the mechanism underlying the induction of tolerance remains unknown.

MicroRNAs (miRNAs), a recently discovered small molecule family, can regulate the gene expression at the posttranscriptional level^8^,^9^. The most outstanding characteristic of miRNAs is that their sequence is included in their self-genome, different from that observed in small interfering RNA^10^,^11^. Therefore, with respect to innovative drug development, miRNA treatment is not thought to react to living tissue. Because miRNAs regulate the expression of various genes^9^, miRNA therapy is expected to improve various genes related to one disease simultaneously by targeting disease-specific miRNAs, rather than one specific gene or protein. Current research has focused on the relationship between cancer and miRNAs, the study of which has most advanced in this field. On the other hand, a relationship between immunology and miRNAs has been reported^12^,^13^.

In murine liver transplantation models, the allografts are accepted across major histocompatibility complex (MHC) barriers without the use of immunosuppression. The degree of lymphocyte infiltration was seen at the early time point after transplantation, then gradually diminished spontaneously. The pathological score, liver function parameters in the serum and levels of pro- and anti-inflammatory cytokines in the serum increased after hepatic allografting, and peaked at 14 days. However, they subsequently decreased gradually, and all indices returned to within the normal levels 100 days after transplantation without the use of immunosuppressive drugs. These results suggest that the alloimmune response began after grafting; however, some mechanism(s) inhibited the proliferation of infiltrating cells and inflammation. Thereafter, on day 14 after transplantation, this immune regulatory mechanism(s) induced and maintained tolerance for a long period. Thus this model is an appropriate model for clarifying the mechanism(s) underlying rejection and the induction of tolerance. In our present study, we focused on the relationship of the miRNA expression during rejection and tolerance following murine hepatic grafting and found that some miRNAs are related to the induction of tolerance.

## Results

### Spontaneous acceptance of hepatic allografts

The livers of the BR mice were orthotopically transplanted into the allogeneic D2 mice without the use of any immunosuppressant agents. The survival of the recipients was monitored. All of the BR livers transplanted into the D2 mice exhibited indefinite survival (MST > 100 days; n = 12).

### Monitoring of the histological findings and functional assessment of the liver allografts

**Figure 1:**
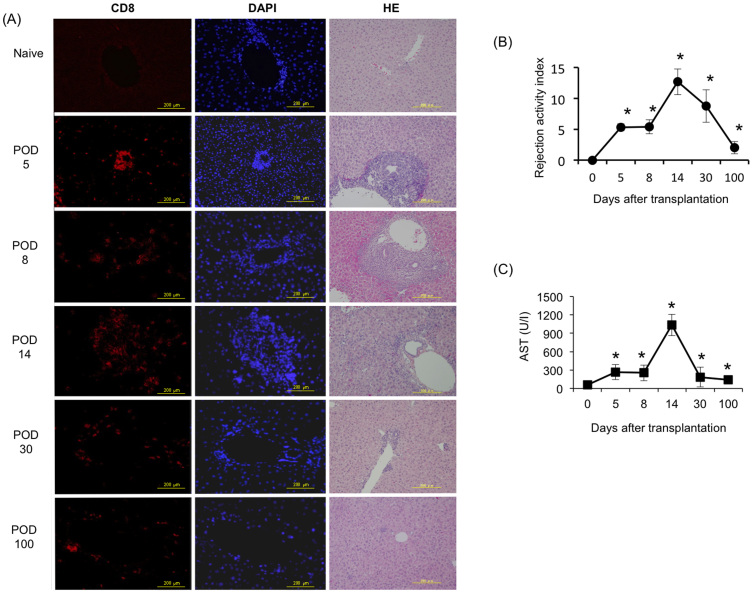
Analysis of pathological cytotoxic T lymphocyte (CTL) infiltration and a function assessment in the mouse hepatic allograft model. BR livers were transplanted into D2 mice. The pathological lesions were monitored on days 0, 5, 8, 14, 30 and 100 posttransplant. (A) CD8^+^ CTLs infiltration was dramatically increased in the grafts. HE staining revealed extensive hemorrhaging and/or parenchymal necrosis on days 5, 8 and 14. (POD = post-operative day; Scale bars represent 200 μm). (B) The severity was graded according to the Banff liver rejection criteria. (C) The AST level was significantly elevated on days 5 and 8 after hepatic transplantation and peaked on day 14 posttransplant. The AST level was then obviously and quickly downregulated on days 30 and 100 posttransplant. All experiments, data were analyzed of three mice per each time point and expressed as the mean ± standard deviation (SD), except for the levels of AST in day30, which reflect the average values from two mice. *p < 0.05 compared with day 0.

The spontaneous mouse liver tolerance model exhibited rejecting injury that peaked on day 14. On day 30, posttransplant tissue repair and remodeling were found. By day 100, the posttransplant tissues reverted back to a normal liver architecture, as determined by an examination of H&E staining using paraffin sections (Fig. 1A). To monitor the progression of rejection, an allograft histological analysis was performed on days 5, 8, 14, 30 and 100 postallograft and scored according to the Banff schema grading system. The RAI on days 5 and 8 posttransplant was 5.3 ± 0.58 and 5.4 ± 1.14, respectively, and the grade was moderate. The RAI on day 14 posttransplant reflected the peak of rejection (12.7 ± 2.24), and increased CD8+ lymphocyte infiltration marked severe expansion around the portal area (Fig. 1A). Furthermore, TUNEL staining revealed that the programmed cell death was occurred after mice liver allotransplantaion (Supplemental Fig. 1). Thereafter, on day 30 posttransplant, the infiltration in the portal vein, bile duct and vein was decreased (8.75 ± 2.62). Then, on day 100 posttransplant, the infiltration was observed to have mostly diminished and the findings were close to those of a normal liver, with an RAI of 0 (Fig. 1B).

Furthermore, to address the liver function after liver transplantation, the serum AST levels were assayed on days 5, 8, 14, 30 and 100 posttransplant. Compared with that observed in the naive mice, the serum AST levels were significantly increased at five and eight days after transplantation (p < 0.05) and peaked at 14 days posttransplant (p < 0.05). By day 30 and 100 posttransplant, the levels of AST had decreased coincident with the resolution of hepatic inflammation, as observed on histology (Fig. 1A, C).

### Serum cytokine assay after liver transplantation

**Figure 2:**
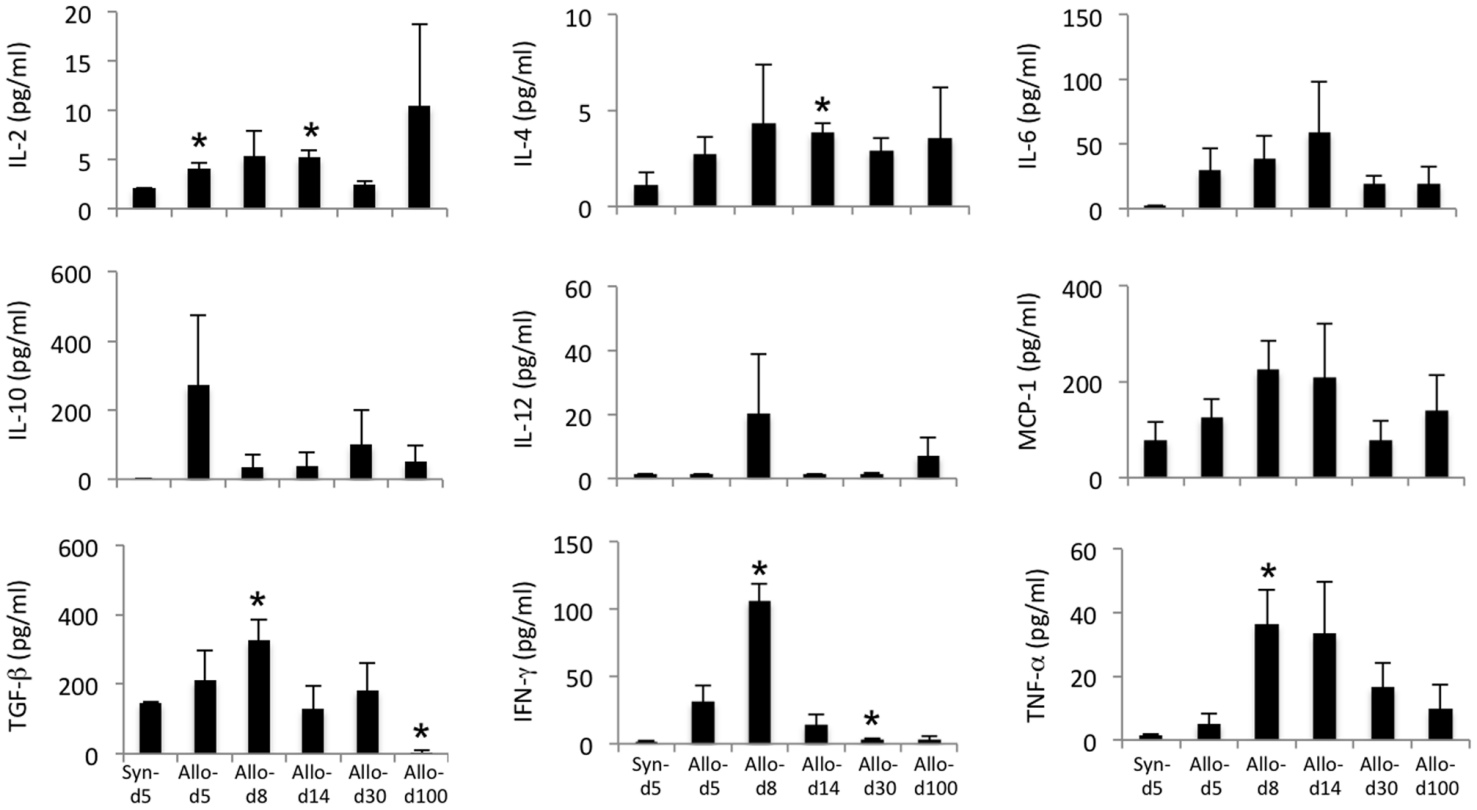
Changes in the levels of serum cytokines in the murine hepatic allografts. To address the changes in the cytokine levels after liver transplantation, the levels of IL-2, IL-4, IL-6, IL-10, IL-12, MCP-1, TGF-β, IFN-γ and TNF-α in the serum on days 5, 8, 14, 30 and 100 postallograft were measured using a BD™ Cytometric Bead Array. All experiments, data were analyzed of three mice per each time point and expressed as the mean ± standard error of the mean (SEM), except for day5 and 8, which was analyzed by five mice. *p < 0.05 compared with day 0.

To investigate the cytokine production in the serum following hepatic transplantation, we measured the levels of IL-2, IL-4, IL-6, IL-10, IL-12, MCP-1, TGF-β, IFN-γ and TNF-α on days 5, 8, 14, 30 and 100 postallograft using a BD Cytometric Bead Array (Fig. 2). The levels of IL-2 and T-cell derived cytokines peaked on day 14. The levels of IL-6, IFN-γ and TNF-α, proinflammatory cytokines, and IL-4 and TGF-β, anti-inflammatory cytokines, peaked on day 8 or day 14 and then decreased until day 100. The level of MCP-1, a monocyte chemotactic protein, also peaked on day 14. This tendency was consistent with that observed for cell infiltration in the grafts, which peaked after transplantation.

### miRNA expression profiles of the murine hepatic allografts

**Figure 3:**
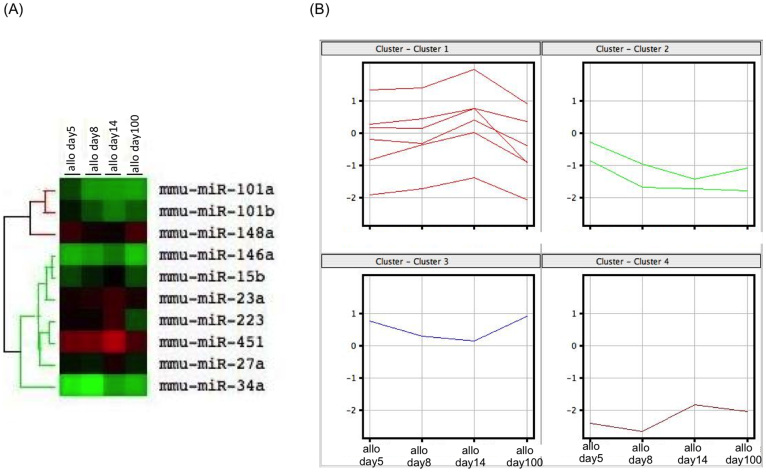
The differential expression of the miRNAs was monitored on days 5, 8, 14 and 100 postallograft compared with that observed in the syngeneic grafts on day 5. (A) A heat map representation of the differentially regulated miRNAs in the hepatic allografts at different time points after hepatic transplantation compared with that observed in the syngeneic grafts on day 5. The progressively brighter shades of red indicate a gradual increase in the miRNA concentration, whereas the shades of green indicate a gradual decrease in the miRNA expression levels. The expression levels ranged from -3-fold to 3-fold. A miRNA clustering tree is shown on the left. The microarray results were hierarchically clustered using the GeneCluster program. (B) A cluster analysis of the 10 miRNAs showed a >3-fold change between day 5 after syngeneic grafting and day 14 after allograft transplantation (p < 0.05). Clusters 1 and 4 contain miR-146a, 15b, 23a, 223, 451 and 27a, and miR-34a, respectively. Clusters 2 and 3 contain miR-101a and 101b, and 148a, respectively. All experiments, data were analyzed of three mice per each time point.

The analysis of the miRNA expression was performed by microarray. All microarray data were registered into the National Center for Biotechnology Information's Gene Expression Omnibus database (http://www.ncbi.nlm.nih.gov/geo/) with the accession number of GSE52164. The heat map of all miRNA analyzed by Agilent miRNA microarrays was shown in Supplementary Figure 2. According to the miRNA microarray analysis, we identified that 10 miRNAs from liver allograft exhibited at least at one time point in time course (day 5, 8, 14, 100 post-transplantation), 3-fold lower or higher expression values compared with syngeneic liver graft on day 5 post-transplantation. The signal intensity of these 10 miRNAs showed significant difference compared with syngeneic liver graft on day 5 post-transplantation (p < 0.05). A hierarchical clustering analysis of the miRNA expression profiles differentiated the time point of hepatic allografting following transplantation from the syngeneic grafts to be day 5 (Fig. 3A). The cluster had two groups, one consisting of miR-101a, 101b and 148a and the other consisting of miR-146a, 15b, 23a, 223, 451, 27a and 34a (Table 1). According to Figure 3B, the former miRNAs were downregulated compared with that observed in the syngeneic grafts, and the expression intensity generally gradually decreased after transplantation, hitting the bottom on day 14. Then, the expression intensity increased on day 100, although it remained downregulated compared with that observed in the syngeneic grafts. In contrast, all of the expression levels in the latter cluster increased after transplantation and peaked on day 14, then decreased until day 100. The raw microarray data files have been deposited in the Gene Expression Omnibus repository (GSE52164).

### Changes in the miRNA expression after liver transplantation

**Figure 4:**
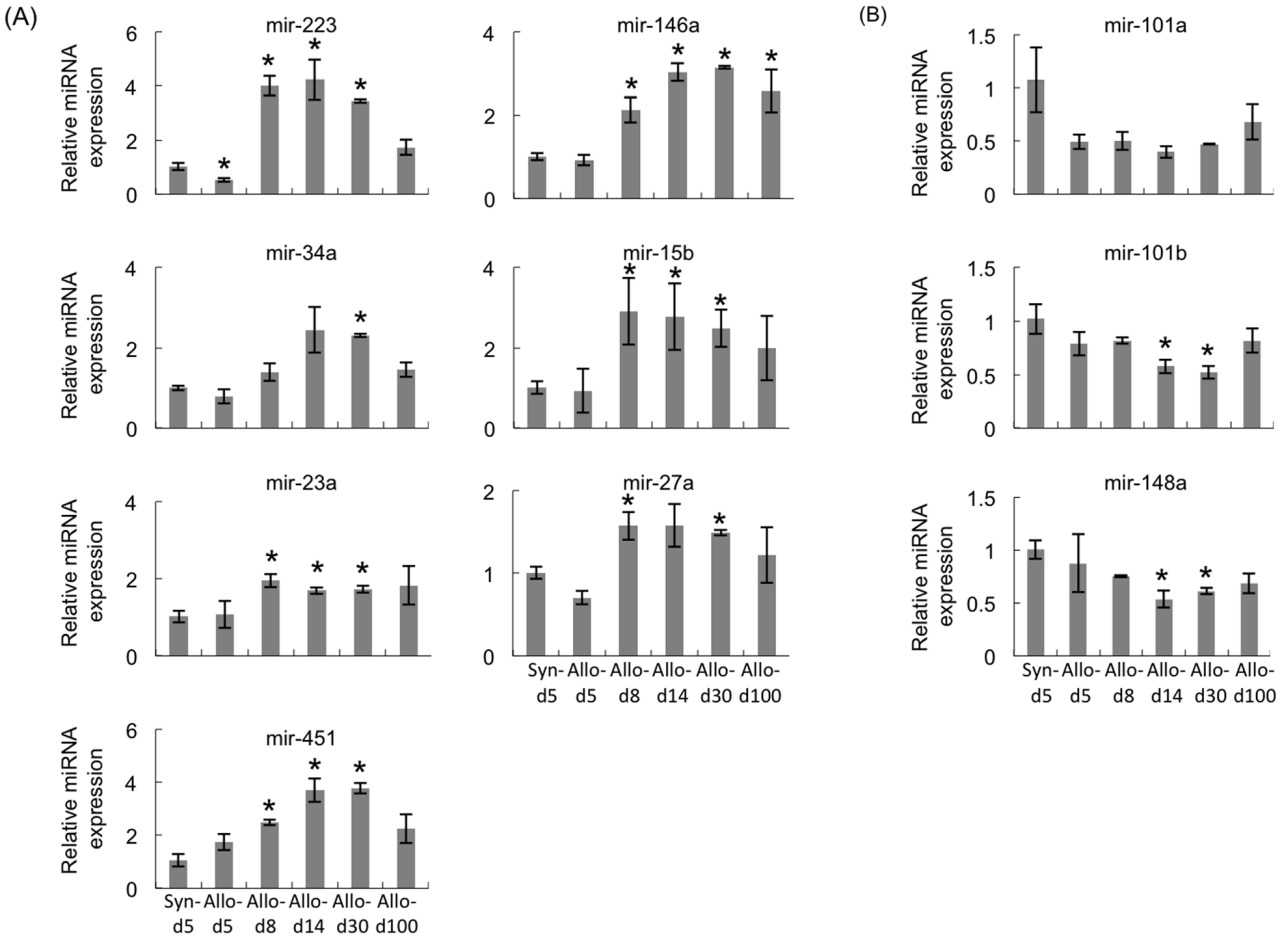
Validation of the microarray data using a real-time RT-PCR assay in the mouse hepatic graft model. The miRNA levels were analyzed on days 5, 8, 14, 30 and 100 in the hepatic allografts. The graph indicates the number of copies of each of the three representative miRNAs measured in the syngeneic grafts or allografts obtained from three individuals. The relative amount of each miRNA was normalized to that of U6 snRNA. (A). The expression levels of seven miRNAs were upregulated. (B). The expression levels of three miRNAs were downregulated according to the pathological conditions after hepatic allografting. All experiments, data were analyzed of three mice per each time point and expressed as the mean ± SEM, except for day30, which reflect the average values from two mice. *p < 0.05 compared with syngeneic day 5.

To confirm the changes in the expressions of the 10 miRNAs that showed a difference from the syngeneic grafts using a miRNA microarray, quantitative RT-PCR was performed. Consistent with the results of the microarray, the expressions of miR-23a, 15b, 27a, 146a, 34a, 451 and 223 were consistent with the level of inflammation (Fig. 4A), and the changes in the expressions of miR-101a, 101b and 148a were in inverse proportion to the inflammation level after transplantation (Fig. 4B).

### Correlations between the graft miRNA levels and the pathological scores and function

**Figure 5:**
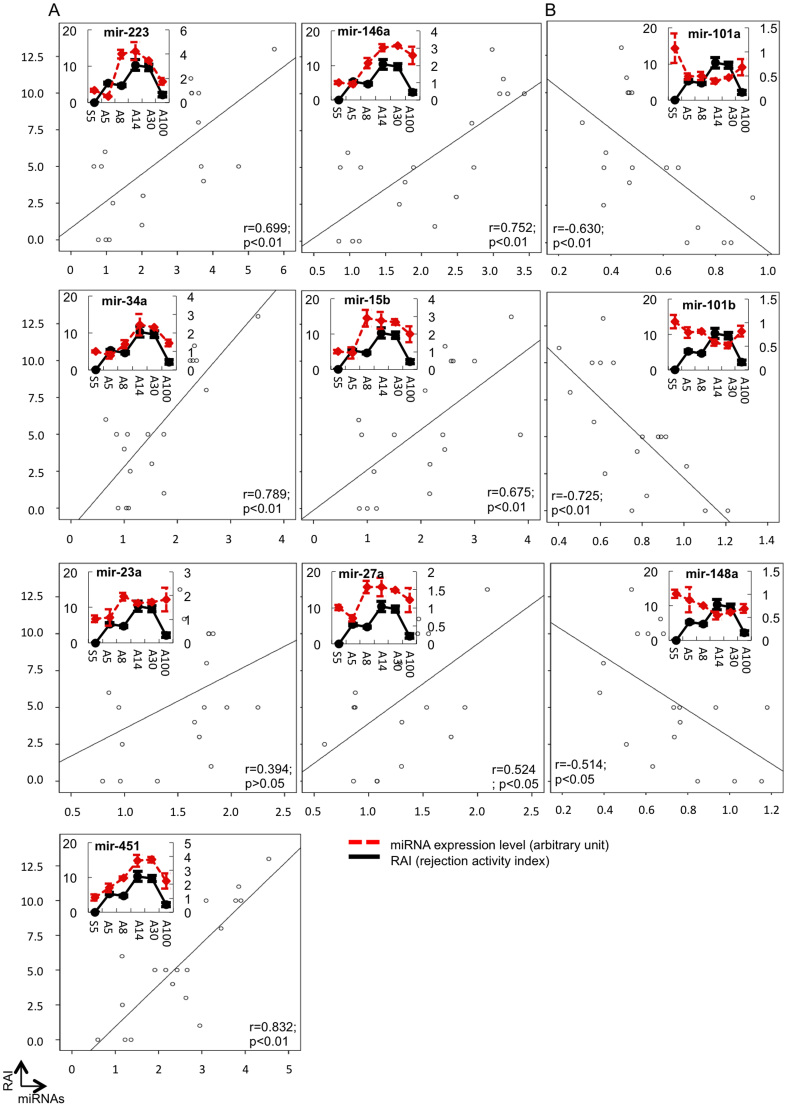
Expression assessment of 10 miRNAs in the liver grafts using a real-time RT-PCR analysis system and an analysis of the relationship between the miRNA levels in the grafts and the pathological scores. The miRNA expression levels were analyzed as described in Figure 4. Pearson's correlation analysis was used to estimate the relationship between the gene expression and the rejection activity index. (A). The expression levels of seven miRNAs were upregulated. (B). The expression levels of three miRNAs were downregulated according to the pathological conditions after hepatic allografting. All experiments, data were analyzed of three mice per each time point and expressed as the mean ± SEM, except for day30, which reflect the average values from two mice.

**Figure 6:**
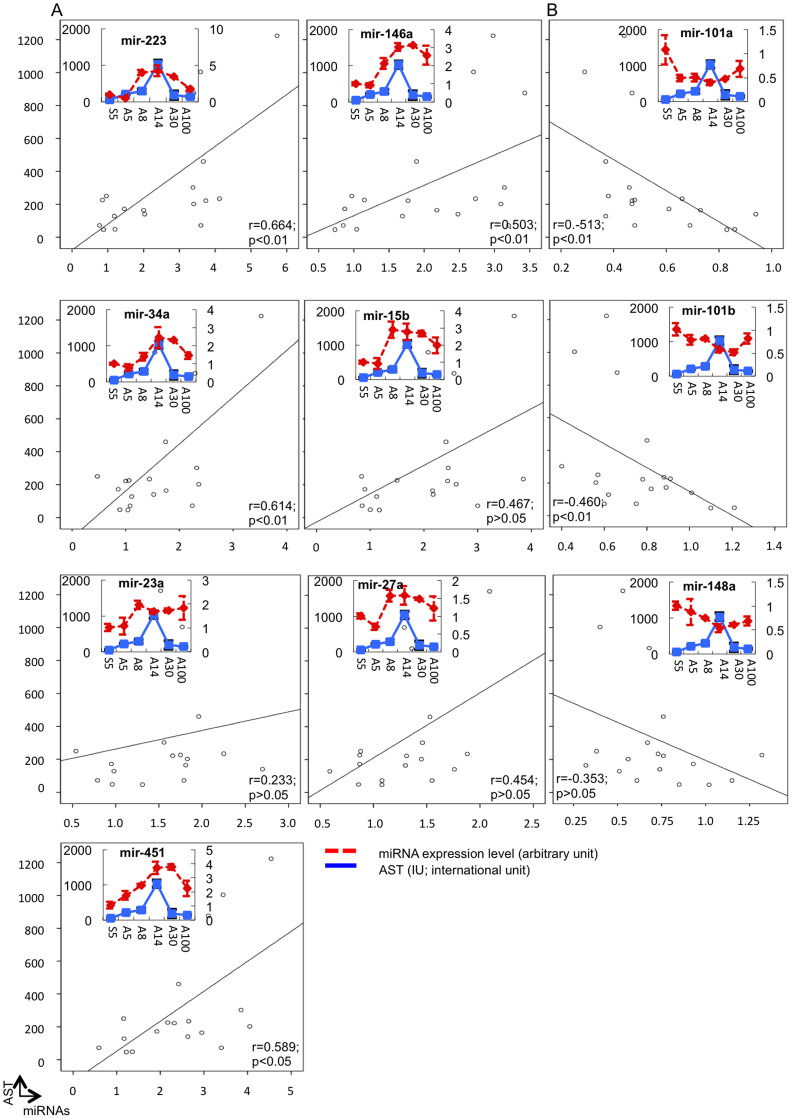
Expression assessment of 10 miRNAs in the liver grafts using a real-time RT-PCR analysis system and an analysis of the relationship between the miRNA levels in the grafts and the function parameters. The miRNA expression levels were analyzed as described in Figure 4. Pearson's correlation analysis was used to estimate the relationship between the gene expression and the AST level. (A). The expression levels of seven miRNAs were upregulated. (B). The expression levels of three miRNAs were downregulated according to the pathological conditions after hepatic allografting. All experiments, data were analyzed of three mice per each time point and expressed as the mean ± SEM, except for day30, which reflect the average values from two mice.

**Figure 7:**
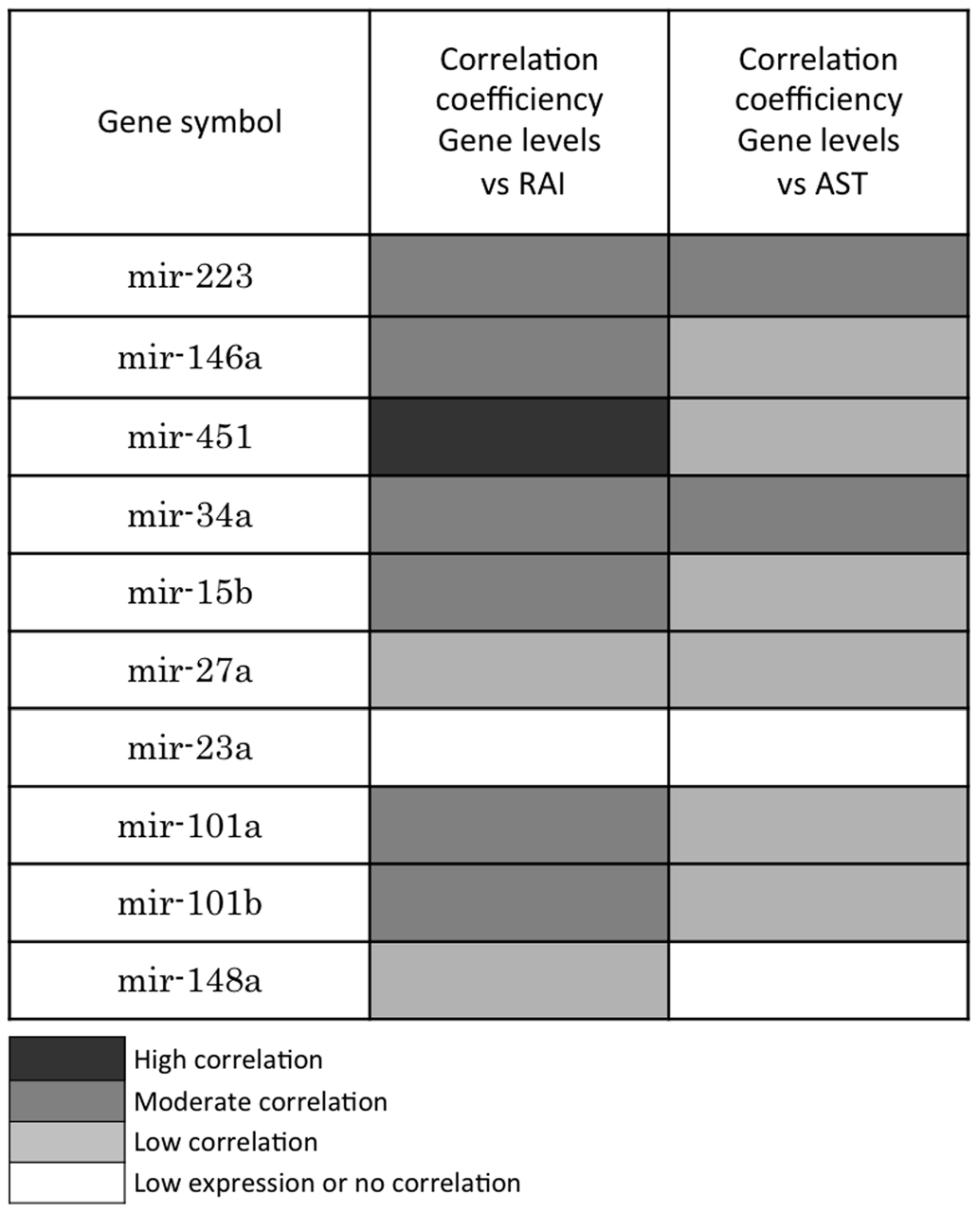
Relationships between the expression levels of the 10 miRNAs in the liver grafts and the pathological scores and function parameters. A color legend is shown as follows: Genes with a highly significant correlation (r = 0.8 ~ 1.0; p < 0.01) relative to the pathological score (RAI)/AST are depicted in dark gray, those with a moderately significant correlation (r = 0.6 ~ 0.8; p < 0.01) are depicted in gray and those with a relatively low significant correlation are depicted in slight gray (r = 0.4 ~ 0.6; p < 0.05). Genes with no significant correlation are depicted in white (p > 0.05).

To examine whether any relationships existed between the levels of the 10 miRNAs in the hepatic grafts and the pathological scores and function, Pearson's correlation analysis was performed. As shown in Figure 5 and 6, positive associations between the expressions of seven miRNAs, miR-223, 23a, 15b, 27a, 146a, 34a and 451, and both the pathological findings (Fig. 5A) and liver function (Fig. 6A) were consistent with the level of inflammation. The expression levels of the seven miRNAs increased, reaching a peak 14 days after transplantation and subsequently decreasing. In contrast, the levels of the three residual miRNAs (miR-101a, 101b and 148a) decreased after transplantation, reaching the lowest level 14 days after transplantation then increasing (Fig. 5B and 6B). We classified the level of correlation as high (r = 0.8 ~ 1.0, p < 0.01), moderate (r = 0.6 ~ 0.8, p < 0.01), low (r = 0.4 ~ 0.6, p < 0.05) or no correlation (p > 0.05) (Fig. 7). The results suggest that miR-223, 146a, 451, 34a and 15b are strongly correlated with inflammation, while miR-101a and 101b are correlated with the induction or maintenance of tolerance.

## Discussion

In order to investigate the genetic mechanisms underlying the spontaneous induction of tolerance, we isolated and analyzed miRNAs in syngeneic and allogeneic graft tissue using an miRNA microarray. In this study, we identified 10 miRNAs in the hepatic allografts that exhibited a difference compared with that observed in the syngeneic grafts (Table 1). Seven of these 10 miRNAs were upregulated (miR-223, 146a, 15b, 23a, 27a, 34a and 451) and three were downregulated (miR-101a, 101b and 148a) compared with that observed in the syngeneic grafts. The former seven miRNAs exhibited positive correlations between their expression level and the immune response reflected by the levels of inhibitory cytokines. This suggests that the upregulated miRNAs were correlated with inflammation and/or the inhibitory response. In our experiment, the kinetic of AST and RAI demonstrated that day 14 after liver transplantation is the peak of inflammatory response and immunoreaction against graft, whereas the production of IL-2 and IL-4 after liver transplantation was somewhat lower concentration in the previously published data^14^,^15^.

In the up-regulated miRNAs, miR-223 is highly expressed in a variety of organs^16^,^17^ and cells^18^. Together with other miRNAs, miR-223 fine-tunes the differentiation of myeloid precursors into either granulocytes or monocytes^19^,^20^ and regulates the differentiation and activation of these cells^21^. Moreover, it has been reported that miR-223 regulates the differentiation of myeloid-derived suppressor cells (MDSCs)^22^ that contribute to cancer evasion from the immune system via their high potential for suppressing the immune response^23^. We found that MDSCs are significantly upregulated in tolerant hepatic allografts (unpublished data). The overexpression of miR-223 in MDSCs is associated with slower tumor growth in mice with reconstituted subcutaneous tumors^22^. This suggests that miR-223 contributes to immune suppression by regulating the inhibitory system of immune responses of immune suppressive cells, such as MDSCs. MiR-223 is overexpressed in peripheral blood nuclear cells in renal allografted patients with acute rejection compared to that observed in those without acute rejection^24^. Furthermore, miR-223 level is highly predictive of acute rejection and strongly linked to the intragraft expression of CD3 mRNA^25^. The level of miR-223 is significantly higher in liver allografts with acute rejection in rats^26^. These previous studies highlight the important role of miR-223 in allograft rejection and the potential use of the miR-223 level as a biomarker of allograft rejection.

In the present study, miR-146a, another upregulated miRNA, exhibited a higher expression level in the tolerogenic allografts (day 100 after transplantation) than in the syngeneic grafts in the hepatic allografts. This data suggest that, to some extent, the role of miR-146a is to maintain the tolerogenic status. The macrophage inflammation responses to microbial infection involve the upregulation of miR-146^27^. The expression of miR-146a/b is nuclear factor-kappa B (NF-κB)-dependent and that the targets of miR-146a/b include TNF receptor-associated factor 6 (TRAF6) and IL-1 receptor-associated kinase 1 (IRAK1), which are key adapter molecules downstream of Toll-like receptors (TLR)s and cytokine receptors. Therefore, the authors proposed a role for miR-146a/b in controlling TLR and cytokine signaling through a negative feedback regulation loop involving the downregulation of the IRAK1 and TRAF6^27^. On the other hand. in accordance with our data for the liver allografts, miR-146a is highly expressed in renal rejecting allografts^24^, with a low expression is on naïve T-cells. MiR-146 exhibits a high expression level on Th1 cells, but not on Th2 cells, relative to the acceleration of Th1 cytokines, thus suggesting that Th1-specific miRNA is related to the acceleration of Th1 cytokines in the immune response^28^. The expression of miR-146a was significantly upregulated after allograft transplantation with acute rejection compared with that observed using a syngeneic combination in a rat liver transplantation model^26^. Taken together, these data suggest that miR-146a correlated with rejection and plays a certain role in the induction and/or persistence of tolerance via the negative feedback regulation of inflammatory responses.

MiR-34a, previously known for its potent tumor suppressive role, is a novel regulator of inflammation. The expression of miR-34a was downregulated in macrophages after lipopolysaccharide (LPS) stimulation. MiR-34a mimics decreased the expression of inflammatory cytokines, such as TNF-α and IL-6, in LPS-treated RAW264 cells, while the inhibition of mrR-34a increased their expression. Notch1 expression was downregulated by miR-34a, and the knockdown of Notch1 exhibited similar effects as miR-34a mimics on the LPS-induced macrophage inflammatory response. The NF-κB activation by LPS was also significantly suppressed by miR-34a^29^. These results suggest that miR-34a is a negative regulator of LPS-induced inflammation, and that this occurs at least partially as a result of its targeting Notch1. In contrast, miR-34a is involved in the downregulation of SIRT1 activity, and it has been reported that the inhibition of SIRT1 disrupts the stimulation of the NF-κB-induced inflammatory responses in chronic metabolic and age-related diseases^30^,^31^. These reports demonstrated that miR-34a might have proinflammatory properties.

A significant increase of the expression of miR-15b and miR-451 was observed in C57BL/6 mice that received intraperitoneal injections of LPS^32^. In addition, in acute liver failure, inhibition of miR-15b reduced the hepatic apoptosis via BCL2 regulation and TNF-α production^33^. These data suggest that miR-15b regulates TNF-α-mediated hepatic apoptosis via BCL2 during acute liver failure. Furthermore, the expression of miR-15b was found to be significantly elevated in the patients with fatty liver disease^34^. These data demonstrated that miR-15b and miR-451 are involved in the development of liver inflammation.

The miR-223, miR-23a and miR-15b levels were downregulated in the sera of multiple sclerosis patients. Further, the expression levels of miR-223 and miR-23a were altered in PBMCs from multiple sclerosis patients^35^. Additionally, miR-23a was expressed at significantly higher levels in the blood from patients with Crohn's disease^36^. MiR-23a was demonstrated to be induced by treatment with baicalin, a flavonoid, consistent with the attenuation of skin damage by ultraviolet radiation B^37^. These data suggested that miR-23a could play a role in the pathogenesis of neural disorders and have some beneficial effects for treating damaged skin.

MiR-27a and miR-23a belong to the same cluster. In an *in vitro* experiment using RAW264 macrophages, the inflammatory response to LPS stimulation showed that the expression of miR-27a was lower in treated cells than in the untreated control cells^38^. In addition, the treatment of the cells with hydrogen peroxide led to a reduction of the miR-27a expression^39^. Furthermore, the expression of miR-23a and miR-27a/b was significantly lower in the mouse livers damaged by carbon tetrachloride administration than in the normal liver^40^. Therefore, miR-27a, as well as miR-23a, suppressed the infiltration of CXCR4-positive inflammatory cells by targeting the CXCL12 gene^40^,^41^,^42^. These previous reports suggested that miR-27a might play a role in inhibiting inflammation.

MiR-451 was reported to act as a “tuner” of gene expression related to erythroid homeostasis^43^. MiR-451 confers protection against simulated ischemia/reperfusion-induced cardiomyocyte death at least partly by targeting the CUG triplet repeat-binding protein 2- cyclooxygenase-2 (COX-2) pathway^44^. In the non-alcoholic fatty liver disease model, the treatment with a dietetic regimen, which caused various liver injuries, demonstrated that there was significant downregulation of miR-451 and miR-27 in the livers of these rats^45^. Furthermore, in a mouse-to-rat cardiac xenotransplantation model, miR-451 in the grafts was lower, while miR-146a was higher than the isograft control at the endpoint of rejection^46^. These data suggest that miR-451 is involved in the anti-inflammatory response(s).

With regard to the downregulated miRNAs, miR-101a regulates the activation of mitogen-activated protein kinase (MAPK) in macrophages by targeting MAPK phosphatase-1^47^. The induction of liver damage in mice exposed to 2,3,7,8-tetrachlorodibenzo-p-dioxin (TCDD) showed that miR-101a was differentially downregulated by TCDD. Further, the targets of miR-101a, such as COX-2, enhancer of zeste homolog 2 and cFos, were all upregulated. These data demonstrated that miR101a plays a significant role in the development of liver damage in mice exposed to TCDD^48^. The miRNA expression levels in splenic lymphocytes from murine lupus models revealed that miR-101a was also markedly upregulated in splenic T cells, but not B cells, from MRL-lpr mice^49^. MiR-101a is also upregulated in CD4 T cells from sanroque mice, which develop lupus-like autoimmune syndrome as a result of the loss of Roquin-mediated repression of the inducible T-cell co-stimulator (ICOS)^50^. In addition, in a xenotransplantation model using SCID mice, miR-101-overexpressing xenograft tumor tissues showed decreased capillary densities and decreased levels of vascular endothelial growth factor (VEGF) and COX-2 by directly targeting them^51^,^52^,^53^. The development of colonic inflammation in IL-10 knockout mice was accompanied by the upregulation of miR-101, miR-223 and miR-146a. *In vitro* exposure of colonic intraepithelial lymphocytes to IL-10 resulted in the downregulation of miR-101 and miR-223^54^. These reports suggested that miR-101a plays an important role in the anti-inflammatory responses.

C57BL/6 mice infected with self-healing *Plasmodium chabaudi* malaria develop protective immunity. This infection induces an upregulation of hepatic mRNAs encoding inflammatory cytokines. MiR-101b is downregulated in this infection, and the downregulation of the miR-101b is retained in mice with protective immunity, and is even sustained upon homolog re-infections of immune mice. These data suggest that miR101b is involved in the development of protective immunity. Furthermore, the tissues from mice deficient in E2F1, a transcription factor involved in the regulation of the inflammatory response to TLRs, treated with systemic LPS revealed that the E2F1-deficient mice showed significantly different regulation of miR-101b compared with wild type controls. These data suggested that miR-101b is involved in the innate immune response to systemic LPS^55^. In addition, high mobility group A1 (HMGA1) protein, a positive inducer of inflammatory signals, positively regulates the expression of miR-101b^56^, further suggesting that miR-101b is involved in the inflammatory responses.

The hepatocyte-derived miRNA analysis of the serum samples from healthy controls and liver transplant recipients and peritransplant liver allograft biopsy samples demonstrated that the expression of miR-148a in the liver tissue was significantly reduced by prolonged graft warm ischemia times^57^. The levels of miR-148a closely correlate with the AST and ALT levels during posttransplant liver injury and acute rejection. This study showed that miR-148a is involved in liver injury. The strong TCR signaling, which is elicited by high affinity ligands or by extended ligand exposure, inhibits foxp3 expression in conventional T cells at the epigenetic level. The suppression of miR-148a by strong TCR signal leads to depression of DNMT1 mRNA translation, which then modulates the expression of foxp3 epigenetically^58^. MiR-148a downregulates HLA-G expression by binding its 3′UTR, and this downregulation of HLA-G affects leukocyte immunoglobulin-like receptor subfamily B member 1 (LILRB1) recognition, and consequently abolishes the LILRB1-mediated inhibition of NK cell killing. In the placenta, miR-148a is expressed at relatively low levels, compared to that in other healthy tissues, and the mRNA levels of HLA-G are particularly high in the placenta, suggesting that this might enable the tissue-specific expression of HLA-G^59^. These studies demonstrated that miR-148a has a major role in immune regulation.

In this experiment, several miRNA day 100 after transplantation also expressed on day 14, irrespective of big difference of the AST and RAI. The reason why is at least there are two mechanisms of immunological reaction in the day 14. On day 14 after liver transplantation, the histology and AST are very severe, which demonstrated that proinflammatory immunological response in the grafted liver. However, coincidentally the mechanisms of induction and maintenance tolerance might be also already active. Therefore, the miRNA expressed on day 100 after liver transplantation also expressed on day 14.

The miRNAs are generated at high amounts in cells with tissue specificity^60^,^61^ and have recently been shown to be strikingly stable in plasma^62^,^63^. In our results, we identified several candidate miRNAs that are possible biomarkers for monitoring the induction of tolerance, although these miRNAs were detected in the graft not the plasma. In order to easily monitor the induction of tolerance, it is better to use plasma as a specimen to detect miRNAs after liver transplantation. Therefore, further studies identifying miRNAs in the plasma during tolerance induction are needed, as recently described using an acute rejection model of rat liver transplantation^26^ and patient samples^57^. Farid et al.^57^ chose 15 miRNAs found on the bibliography and examined the levels of expression in the serum of affected individuals after liver transplantation. Their outcomes suggest that miRNAs derived from hepatocytes are potential candidates as early, stable and sensitive biomarkers of rejection and hepatic injury following liver allografting. Furthermore, Hu et al.^26^ demonstrated the global miRNA expression profiles in rat plasma and hepatic allografts. In their results, nine miRNAs exhibited significant changes in both the plasma and graft. Eight of the nine miRNAs demonstrated reciprocal changes in the plasma and graft.

It is easy to induce tolerance in liver allografts compared with other organs; however, the mechanism underlying this phenomenon remains unknown. With respect to the regulation of immunosuppressive drugs, identifying biomarkers of tolerance is useful. In this study, we found that some miRNAs are correlated with the suppressive response using a unique mouse liver transplantation model. Our results indicate that monitoring the graft expression of novel miRNAs may allow clinicians to differentiate between rejection and tolerance.

## Methods

### Animals

B10.BR (BR, H2-K^k^) and B10.D2 (D2, H2-K^d^) male mice weighing 25–30 g were purchased from the Shizuoka Laboratory Animal Center (Shizuoka, Japan). All mice were maintained under standard conditions and fed rodent food and water, in accordance with the guidelines of the Animal Use and Care Committee of the National Research Institute for Child Health and Development. All animal experiments were performed according to the recommendations of the Committee on the Care and Use of Laboratory Animals at the National Research Institute for Child Health and Development. The protocol was approved by the Committee on the Care and Use of Laboratory Animals at the National Research Institute for Child Health and Development (Permit Number: 2002-003). All surgery was performed under ether anesthesia, and all efforts were made to minimize suffering.

### Orthotopic liver transplantation

BR mice were used as donors and D2 mice were used as recipients. We performed transplantation surgery on the mice as previously described^6^. In brief, after flushing 1 ml of cold saline through the portal vein, the donor liver was removed and preserved in the same solution. We subsequently transplanted the livers into the recipient mice using the cuff technique.

### Histological studies

For the histological observations, three mice from each group were sacrificed, and the liver grafts were removed on days 5, 8, 14, 30 and 100 after transplantation. Liver tissues fixed in 10% phosphate-buffered formalin were embedded in paraffin, and 5-μm-thick sections were stained with hematoxylin and eosin for standard microscopy. The histological findings were graded according to the Banff schema with modification (Table 2) to grade hepatic allograft rejection^64^, and the rejection activity index (RAI) was defined and used to grade acute rejection. Specimens measuring up to 1 cm^3^ were embedded in OCT compound (Tissue-Tek, Elkhart, IN) and stored at −80°C for immunohistochemical staining using anti-CD8 monoclonal antibodies (BD PharMingen, San Diego, CA), as previously described^5^. To detect Programed cell death, a TUNEL assay was performed on paraffin-embedded sections using the ApopTag Plus peroxidase in situ apoptosis detection kit (CHEMICON, Billerica, MA) according to the manufacturer's instructions.

### Measurement of the serum AST levels

The serum aspartate aminotransferase (AST) levels were used as indicators of hepatic dysfunction. The assays were performed with mice serum obtained after liver transplantation using a GOT/AST-PIII diagnostic kit (Fujifilm, Kanagawa, Japan), as described by the manufacturer.

### RNA isolation from hepatic graft tissue

Total RNA, including miRNA from RNAlater (Ambion, Austin, TX) immersed liver grafts, was isolated using the RNeasy Mini Kit and miRNeasy kit (QIAGEN Inc., Valencia, CA.), according to the manufacturer's protocol. The quality and quantity of the RNA were evaluated using the NanoDrop 1000 (Thermo Scientific Inc., Waltham, MA) and Agilent 2100 Bioanalyzer (Agilent Technologies, Santa Clara, CA).

### miRNA array hybridization and data analyses

One hundred ng of total RNA was dephosphorylated with calf intestinal alkaline phosphatase (Takara Bio Inc., Shiga, Japan), followed by denaturing with heat in the presence of dimethyl sulfoxide (SIGMA-Aldrich Co., St. Louis, MO). A cyanine dye, cyanine3-cytidine bisphosphate (Agilent), was then joined to the dephosphorylated single-stranded RNA using T4RNA ligase (Ambion). MicroBioSpin 6 columns (Bio-Rad Laboratories Inc., Hercules, CA) were used to remove any unincorporated cyanine dye from the samples. The purified labeled miRNA probes were hybridized to 8 × 15 K mouse miRNA microarrays (Agilent Technologies) in a rotating hybridization oven at 20 rpm for 20 hours at 55°C. After hybridization, the arrays were washed in Gene Expression Wash Buffer 1 with Triton X-102 followed by Gene Expression Wash Buffer 2 with Triton X-102. After washing, all slides were immediately scanned at a resolution of 5 μm with Green-PMT XDR Hi 100%, Lo 5% using an Agilent DNA Microarray Scan (Agilent). The resulting images were quantified using Agilent's Feature Extraction software program. The differentially expressed miRNAs were identified using a standard protocol developed for miRNA gene arrays. To increase the reliability of the data and ease of detection, miRNA species with a hybridization intensity < 1.5 times the average hybridization intensity (mean) were excluded from the analysis. A miRNA clustering analysis was performed with the Hierarchical clustering algorithm provided in the Avadis, ArrayAssist 4.3 software package.

### Cytokine assay

The levels of interleukin (IL)-2, IL-4, IL-6, IL-10, IL-12, monocyte chemoattractant protein (MCP)-1, transforming growth factor (TGF)-β, interferon (IFN)-γ and tumor necrosis factor (TNF)-α in the serum on days 5, 8, 14, 30 and 100 postallograft were quantified using a BD™ Cytometric Bead Array mouse IL-2, IL-4, IL-6, IL-10, IL-12, MCP-1, TGF-β, IFN-γ and TNF-α Flex Kit (BD Biosciences, Franklin Lakes, NJ) according to the manufacturer's protocol.

### Quantitative real-time reverse transcriptase-polymerase chain reaction (RT-PCR)

The relative quantification of the murine miRNA expression was determined using Taqman technology. Primers amplifying the murine pre-miRNA region and a specific Taqman probe were purchased from Applied Biosystems (Foster City, CA). Taqman MicroRNA Assay (Applied Biosystems) technology with a mature miRNA-specific probe and RT and PCR primers was used to quantify mature miRNA, according to the manufacturer's instructions. The amplification of U6 small RNA (Applied Biosystems) was performed with each experimental sample as an endogenous control to account for differences in the amount and quality of total RNA added to each reaction. Each 600 ng of RNA was reverse transcribed to cDNA using an oligo (dT) primer and Super Script™ reverse transcriptase (Invitrogen, Carlsbad, CA) according to the manufacturer's protocol. Quantitative RT-PCR was performed using the TaqMan system on the Applied Biosystems 7900HT Sequence Detection System (Applied Biosystems). Primers amplifying the murine mRNA region and a specific Taqman probe were designed using the PrimerExpress software program (Applied Biosystems). The data are expressed according to the comparative cycle threshold (Ct). The normalized Ct value of each gene was obtained by subtracting the Ct value of 18S rRNA. The fold change versus one sample in the control group was calculated. All primers and the Taqman probe were obtained from Applied Biosystems.

### Statistical analysis

Student's *t*-test was used to compare the paired and unpaired variables. A statistical evaluation of graft survival was performed using the Kaplan-Meier test. *P* values of less than 0.05 were considered to be statistically significant.

## Supplementary Material

Supplementary Information supplementary Figure1&2

## Figures and Tables

**Table 1:** List of microRNAs that differed between the allo and the syngenic liver graft (fold change > 3; p < 0.05)

Upregulated								
miRNA	allo-day5		allo-day8		allo-day14		allo-day100	
Fold change (log_2_)	*p* value	Fold change (log_2_)	*p* value	Fold change (log_2_)	*p* value	Fold change (log_2_)	*p* value	
miR-146a	2.08	0.036	2.38	0.009	3.01	0.008	1.87	0.040
miR-15b	2.92	0.036	4.01	0.010	5.22	0.008	2.78	0.040
miR-223	3.66	0.041	3.62	0.009	5.58	0.008	1.74	0.029
miR-23a	1.73	0.041	1.94	0.015	2.44	0.013	1.83	0.040
miR-27a	1.79	0.036	1.65	0.009	2.70	0.009	1.56	0.023
miR-34a	1.38	0.034	1.18	0.032	2.08	0.045	1.80	0.044
miR-451	2.00	0.027	2.10	0.012	3.14	0.045	1.50	0.042

Downregulated								
miRNA	allo-day5		allo-day8		allo-day14		allo-day100	
Fold change (log_2_)	*p* value	Fold change (log_2_)	*p* value	Fold change (log_2_)	*p* value	Fold change (log_2_)	*p* value	
miR-101a	−1.39	0.034	−2.47	0.015	−2.54	0.030	−2.68	0.040
miR-101b	−1.68	0.048	−2.69	0.010	−3.73	0.012	−2.97	0.015
miR-148a	−1.39	0.042	−1.92	0.009	−2.12	0.012	−1.25	0.039

**Table 2:** Pathological grading of hepatic rejection

Portal Inflammation	0	None
	1	Mostly lymphocytic inflammation involving, but not noticeably expanding, a minority of the triads
	2	Expansion of most or all of the triads, by a mixed infiltrate containing lymphocytes with occasional blasts, neutrophils and eosinophils
	3	Marked expansion of most or all of the triads by a mixed infiltrate containing numerous blasts and eosinophils with inflammatory spillover into the periportal parenchyma
Bile Duct Inflammation Damage	0	None
	1	A minority of the ducts are cuffed and infiltrated by inflammatory cells and show only mild reactive changes such as increased nuclear, cytoplasmic ratio of the epithelial cells
	2	Most or all of the ducts infiltrated by inflammatory cells. More than an occasional duct shows degenerative changes such as nuclear pleomorphism, disordered polarity and cytoplasmic vacuolization of the epithelium
	3	As above for 2, with most or all of the ducts showing degenerative changes or focal lumenal disruption
Venous Endothelial Inflammation	0	None
	1	Subendothelial lymphocytic infiltration involving some, but not a majority of the portal and/or hepatic venules
	2	Subendothelial infiltration involving most or all of the portal and/or hepatic venules
	3	As above for 2, with moderate or severe perivenular inflammation that extends into the perivenular parenchyma and is associated with perivenular hepatocyte necrosis

## References

[b1] Orlando G., Soker S. & Wood K. Operational tolerance after liver transplantation. J Hepatol 50, 1247–1257 (2009). 19394103 10.1016/j.jhep.2009.03.006

[b2] Kawasaki M. *et al.* Gene expression profile analysis of the peripheral blood mononuclear cells from tolerant living-donor liver transplant recipients. Int Surg 92, 276–286 (2007). 18399100

[b3] Calne R. Y. *et al.* Induction of immunological tolerance by porcine liver allografts. Nature 223, 472–476 (1969). 4894426 10.1038/223472a0

[b4] Kamada N., Brons G. & Davies H. S. Fully allogeneic liver grafting in rats induces a state of systemic nonreactivity to donor transplantation antigens. Transplantation 29, 429–431 (1980). 6990572 10.1097/00007890-198005000-00021

[b5] Morita M. *et al.* PD-1/B7-H1 interaction contribute to the spontaneous acceptance of mouse liver allograft. Am J Transplant 10, 40–46 (2010). 19889124 10.1111/j.1600-6143.2009.02859.x PMC2887673

[b6] Morita M. *et al.* Spontaneous tolerance involving natural killer T cells after hepatic grafting in mice. Transpl Immunol 18, 142–145 (2007). 18005859 10.1016/j.trim.2007.05.015

[b7] Xie L. *et al.* Identification of a novel biomarker gene set with sensitivity and specificity for distinguishing between allograft rejection and tolerance. Liver Transpl 18, 444–454 (2012). 22162188 10.1002/lt.22480

[b8] Pasquinelli A. E. *et al.* Conservation of the sequence and temporal expression of let-7 heterochronic regulatory RNA. Nature 408, 86–89 (2000). 11081512 10.1038/35040556

[b9] Pritchard C. C., Cheng H. H. & Tewari M. MicroRNA profiling: approaches and considerations. Nat Rev Genet 13, 358–369 (2012). 22510765 10.1038/nrg3198 PMC4517822

[b10] Fire A. *et al.* Potent and specific genetic interference by double-stranded RNA in Caenorhabditis elegans. Nature 391, 806–811 (1998). 9486653 10.1038/35888

[b11] Rodriguez A., Griffiths-Jones S., Ashurst J. L. & Bradley A. Identification of mammalian microRNA host genes and transcription units. Genome Res 14, 1902–1910 (2004). 15364901 10.1101/gr.2722704 PMC524413

[b12] Baltimore D., Boldin M. P., O'Connell R. M., Rao D. S. & Taganov K. D. MicroRNAs: new regulators of immune cell development and function. Nat Immunol 9, 839–845 (2008). 18645592 10.1038/ni.f.209

[b13] Sonkoly E., Stahle M. & Pivarcsi A. MicroRNAs and immunity: novel players in the regulation of normal immune function and inflammation. Semin Cancer Biol 18, 131–140 (2008). 18291670 10.1016/j.semcancer.2008.01.005

[b14] Li W., Zheng X. X., Kuhr C. S. & Perkins J. D. CTLA4 engagement is required for induction of murine liver transplant spontaneous tolerance. Am J Transplant 5, 978–986 (2005). 15816877 10.1111/j.1600-6143.2005.00823.x

[b15] Shi Y. *et al.* Gene silencing of 4-1BB by RNA interference inhibits acute rejection in rats with liver transplantation. Biomed Res Int 2013, 192738 (2013). 23484089 10.1155/2013/192738 PMC3581255

[b16] Cai P. *et al.* MicroRNA-Gene Expression Network in Murine Liver during Schistosoma japonicum Infection. PLoS One 8, e67037 (2013). 23825609 10.1371/journal.pone.0067037 PMC3692539

[b17] Harraz M. M., Eacker S. M., Wang X., Dawson T. M. & Dawson V. L. MicroRNA-223 is neuroprotective by targeting glutamate receptors. Proc Natl Acad Sci U S A 109, 18962–18967 (2012). 23112146 10.1073/pnas.1121288109 PMC3503176

[b18] Merkerova M., Belickova M. & Bruchova H. Differential expression of microRNAs in hematopoietic cell lineages. Eur J Haematol 81, 304–310 (2008). 18573170 10.1111/j.1600-0609.2008.01111.x

[b19] Haneklaus M., Gerlic M., O'Neill L. A. & Masters S. L. miR-223: infection, inflammation and cancer. J Intern Med 274, 215–226 (2013). 23772809 10.1111/joim.12099 PMC7166861

[b20] O'Connell R. M., Zhao J. L. & Rao D. S. MicroRNA function in myeloid biology. Blood 118, 2960–2969 (2011). 21725054 10.1182/blood-2011-03-291971 PMC3175776

[b21] Johnnidis J. B. *et al.* Regulation of progenitor cell proliferation and granulocyte function by microRNA-223. Nature 451, 1125–1129 (2008). 18278031 10.1038/nature06607

[b22] Liu Q. *et al.* miR-223 suppresses differentiation of tumor-induced CD11b(+) Gr1(+) myeloid-derived suppressor cells from bone marrow cells. Int J Cancer 129, 2662–2673 (2011). 21213211 10.1002/ijc.25921

[b23] Gabrilovich D. I. & Nagaraj S. Myeloid-derived suppressor cells as regulators of the immune system. Nat Rev Immunol 9, 162–174 (2009). 19197294 10.1038/nri2506 PMC2828349

[b24] Anglicheau D. *et al.* MicroRNA expression profiles predictive of human renal allograft status. Proc Natl Acad Sci U S A 106, 5330–5335 (2009). 19289845 10.1073/pnas.0813121106 PMC2663998

[b25] Sui W. *et al.* Microarray analysis of MicroRNA expression in acute rejection after renal transplantation. Transpl Immunol 19, 81–85 (2008). 18346642 10.1016/j.trim.2008.01.007

[b26] Hu J. *et al.* Plasma MicroRNA, a Potential Biomarker for Acute Rejection After Liver Transplantation. Transplantation 95, 991–999 (2013). 23466638 10.1097/TP.0b013e31828618d8

[b27] Taganov K. D., Boldin M. P., Chang K. J. & Baltimore D. NF-kappaB-dependent induction of microRNA miR-146, an inhibitor targeted to signaling proteins of innate immune responses. Proc Natl Acad Sci U S A 103, 12481–12486 (2006). 16885212 10.1073/pnas.0605298103 PMC1567904

[b28] Monticelli S. *et al.* MicroRNA profiling of the murine hematopoietic system. Genome Biol 6, R71 (2005). 16086853 10.1186/gb-2005-6-8-r71 PMC1273638

[b29] Jiang P. *et al.* MiR-34a inhibits lipopolysaccharide-induced inflammatory response through targeting Notch1 in murine macrophages. Exp Cell Res 318, 1175–1184 (2012). 22483937 10.1016/j.yexcr.2012.03.018

[b30] Kauppinen A., Suuronen T., Ojala J., Kaarniranta K. & Salminen A. Antagonistic crosstalk between NF-kappaB and SIRT1 in the regulation of inflammation and metabolic disorders. Cell Signal 25, 1939–1948 (2013). 23770291 10.1016/j.cellsig.2013.06.007

[b31] Lee J. *et al.* A pathway involving farnesoid X receptor and small heterodimer partner positively regulates hepatic sirtuin 1 levels via microRNA-34a inhibition. J Biol Chem 285, 12604–12611 (2010). 20185821 10.1074/jbc.M109.094524 PMC2857134

[b32] Hsieh C. H. *et al.* Whole blood-derived microRNA signatures in mice exposed to lipopolysaccharides. J Biomed Sci 19, 69 (2012). 22849760 10.1186/1423-0127-19-69 PMC3419134

[b33] An F. *et al.* miR-15b and miR-16 regulate TNF mediated hepatocyte apoptosis via BCL2 in acute liver failure. Apoptosis 17, 702–716 (2012). 22374434 10.1007/s10495-012-0704-7

[b34] Zhang Y. *et al.* Upregulation of miR-15b in NAFLD models and in the serum of patients with fatty liver disease. Diabetes Res Clin Pract 99, 327–334 (2013). 23287814 10.1016/j.diabres.2012.11.025

[b35] Ridolfi E. *et al.* Expression and Genetic Analysis of MicroRNAs Involved in Multiple Sclerosis. Int J Mol Sci 14, 4375–4384 (2013). 23439547 10.3390/ijms14034375 PMC3634436

[b36] Paraskevi A. *et al.* Circulating MicroRNA in inflammatory bowel disease. J Crohns Colitis 6, 900–904 (2012). 22386737 10.1016/j.crohns.2012.02.006

[b37] Xu Y., Zhou B., Wu D., Yin Z. & Luo D. Baicalin modulates microRNA expression in UVB irradiated mouse skin. J Biomed Res 26, 125–134 (2012). 23554741 10.1016/S1674-8301(12)60022-0 PMC3597329

[b38] Cheng Y. *et al.* Downregulation of miR-27a* and miR-532-5p and upregulation of miR-146a and miR-155 in LPS-induced RAW264.7 macrophage cells. Inflammation 35, 1308–1313 (2012). 22415194 10.1007/s10753-012-9443-8

[b39] Thulasingam S. *et al.* miR-27b*, an oxidative stress-responsive microRNA modulates nuclear factor-kB pathway in RAW 264.7 cells. Mol Cell Biochem 352, 181–188 (2011). 21350856 10.1007/s11010-011-0752-2

[b40] Lu M. H. *et al.* microRNA-27b suppresses mouse MSC migration to the liver by targeting SDF-1alphain vitro. Biochem Biophys Res Commun 421, 389–395 (2012). 22516754 10.1016/j.bbrc.2012.04.027

[b41] Lewis B. P., Shih I. H., Jones-Rhoades M. W., Bartel D. P. & Burge C. B. Prediction of mammalian microRNA targets. Cell 115, 787–798 (2003). 14697198 10.1016/s0092-8674(03)01018-3

[b42] Ouwehand K. *et al.* CXCL12 is essential for migration of activated Langerhans cells from epidermis to dermis. Eur J Immunol 38, 3050–3059 (2008). 18924211 10.1002/eji.200838384

[b43] Rasmussen K. D. *et al.* The miR-144/451 locus is required for erythroid homeostasis. J Exp Med 207, 1351–1358 (2010). 20513743 10.1084/jem.20100458 PMC2901075

[b44] Zhang X. *et al.* Synergistic effects of the GATA-4-mediated miR-144/451 cluster in protection against simulated ischemia/reperfusion-induced cardiomyocyte death. J Mol Cell Cardiol 49, 841–850 (2010). 20708014 10.1016/j.yjmcc.2010.08.007 PMC2949485

[b45] Alisi A. *et al.* Mirnome analysis reveals novel molecular determinants in the pathogenesis of diet-induced nonalcoholic fatty liver disease. Lab Invest 91, 283–293 (2011). 20956972 10.1038/labinvest.2010.166

[b46] Li C. *et al.* Analysis of intragraft microRNA expression in a mouse-to-rat cardiac xenotransplantation model. Microsurgery 34, 44–50 (2014). 23913343 10.1002/micr.22139

[b47] Zhu Q. Y., Liu Q., Chen J. X., Lan K. & Ge B. X. MicroRNA-101 targets MAPK phosphatase-1 to regulate the activation of MAPKs in macrophages. J Immunol 185, 7435–7442 (2010). 21068409 10.4049/jimmunol.1000798

[b48] Yoshioka W., Higashiyama W. & Tohyama C. Involvement of microRNAs in dioxin-induced liver damage in the mouse. Toxicol Sci 122, 457–465 (2011). 21602190 10.1093/toxsci/kfr130

[b49] Dai R. *et al.* Identification of a common lupus disease-associated microRNA expression pattern in three different murine models of lupus. PLoS One 5, e14302 (2010). 21170274 10.1371/journal.pone.0014302 PMC3000827

[b50] Yu D. *et al.* Roquin represses autoimmunity by limiting inducible T-cell co-stimulator messenger RNA. Nature 450, 299–303 (2007). 18172933 10.1038/nature06253

[b51] Hao Y. *et al.* Enforced expression of miR-101 inhibits prostate cancer cell growth by modulating the COX-2 pathway in vivo. Cancer Prev Res (Phila) 4, 1073–1083 (2011). 21430074 10.1158/1940-6207.CAPR-10-0333 PMC3305792

[b52] Zhang J., Han C., Zhu H., Song K. & Wu T. miR-101 inhibits cholangiocarcinoma angiogenesis through targeting vascular endothelial growth factor (VEGF). Am J Pathol 182, 1629–1639 (2013). 23608225 10.1016/j.ajpath.2013.01.045 PMC3644734

[b53] Tanaka T., Haneda S., Imakawa K., Sakai S. & Nagaoka K. A microRNA, miR-101a, controls mammary gland development by regulating cyclooxygenase-2 expression. Differentiation 77, 181–187 (2009). 19281778 10.1016/j.diff.2008.10.001

[b54] Schaefer J. S., Montufar-Solis D., Vigneswaran N. & Klein J. R. Selective upregulation of microRNA expression in peripheral blood leukocytes in IL-10−/− mice precedes expression in the colon. J Immunol 187, 5834–5841 (2011). 22043014 10.4049/jimmunol.1100922 PMC3221883

[b55] Warg L. A. *et al.* The role of the E2F1 transcription factor in the innate immune response to systemic LPS. Am J Physiol Lung Cell Mol Physiol 303, L391–400 (2012). 22707615 10.1152/ajplung.00369.2011 PMC4587596

[b56] De Martino I. *et al.* Regulation of microRNA expression by HMGA1 proteins. Oncogene 28, 1432–1442 (2009). 19169275 10.1038/onc.2008.495

[b57] Farid W. R. *et al.* Hepatocyte-derived microRNAs as serum biomarkers of hepatic injury and rejection after liver transplantation. Liver Transpl 18, 290–297 (2012). 21932376 10.1002/lt.22438

[b58] Li C., Ebert P. J. & Li Q. J. T cell receptor (TCR) and transforming growth factor beta (TGF-beta) signaling converge on DNA (cytosine-5)-methyltransferase to control forkhead box protein 3 (foxp3) locus methylation and inducible regulatory T cell differentiation. J Biol Chem 288, 19127–19139 (2013). 23687305 10.1074/jbc.M113.453357 PMC3696685

[b59] Manaster I. *et al.* MiRNA-mediated control of HLA-G expression and function. PLoS One 7, e33395 (2012). 22438923 10.1371/journal.pone.0033395 PMC3306401

[b60] Ason B. *et al.* Differences in vertebrate microRNA expression. Proc Natl Acad Sci U S A 103, 14385–14389 (2006). 16983084 10.1073/pnas.0603529103 PMC1599972

[b61] Lagos-Quintana M. *et al.* Identification of tissue-specific microRNAs from mouse. Curr Biol 12, 735–739 (2002). 12007417 10.1016/s0960-9822(02)00809-6

[b62] Wang K. *et al.* Circulating microRNAs, potential biomarkers for drug-induced liver injury. Proc Natl Acad Sci U S A 106, 4402–4407 (2009). 19246379 10.1073/pnas.0813371106 PMC2657429

[b63] Mitchell P. S. *et al.* Circulating microRNAs as stable blood-based markers for cancer detection. Proc Natl Acad Sci U S A 105, 10513–10518 (2008). 18663219 10.1073/pnas.0804549105 PMC2492472

[b64] Banff schema for grading liver allograft rejection: an international consensus document. Hepatology 25, 658–663 (1997). 9049215 10.1002/hep.510250328

