# Supplementary material for: Identification of microRNAs involved in acute rejection and spontaneous tolerance in murine hepatic allografts
Source: Sci Rep. 2014 Oct 17;4:6649. doi: 10.1038/srep06649 (PMC5377586; doi:10.1038/srep06649)

Title:

Identification of microRNAs involved in acute rejection and spontaneous tolerance in murine hepatic allografts

Authors:

Miwa Morita[1, 2, †], Jiajie Chen[1, 3, †], Masayuki Fujino[1, 4], Yusuke Kitazawa[1], Atsushi Sugioka[2], Liang Zhong[3, *] and Xiao-Kang Li[1, *]

Supplementary figure legends

Supplementary Figure 1: Programmed cell death after mice liver allotransplantation. TUNEL staining showed extensive programmed cell death after liver allotransplantation along with extensive inflammation in Figure 1 (POD = post-operative day).

Supplementary Figure 2: Full heat map generated from all miRNA expression data for syngeneic on day5, and allogeneic day5, 8, 14 and 100.

Supplementary Figure 1; Morita M, et al

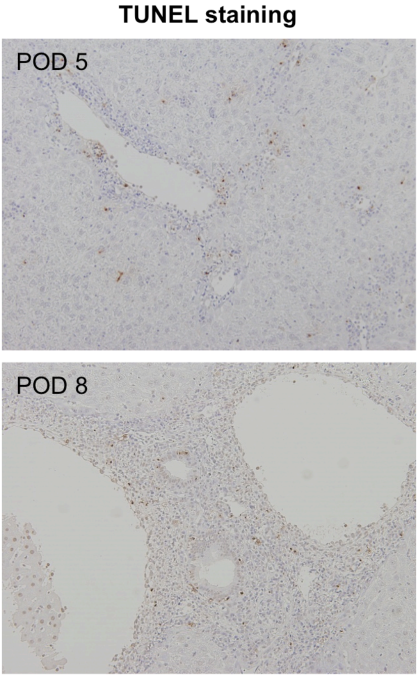

Supplementary Figure 2; Morita M, et al

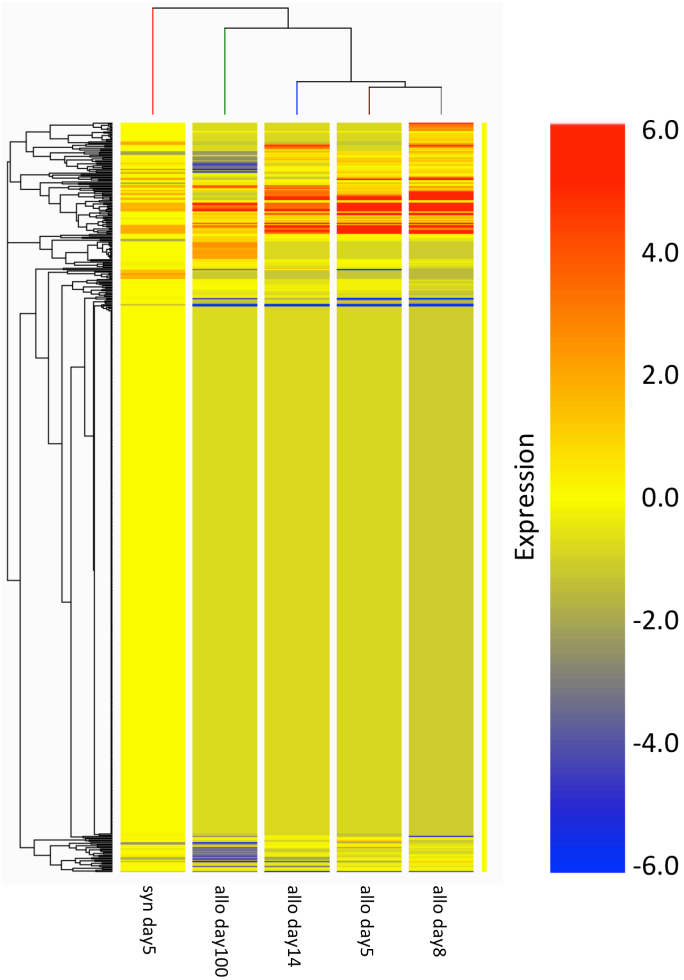

Supplement: Supplementary Information — supplementary Figure1&2 [file srep06649-s1.pdf]
